# Scanning and Filling: Ultra-Dense SNP Genotyping Combining Genotyping-By-Sequencing, SNP Array and Whole-Genome Resequencing Data

**DOI:** 10.1371/journal.pone.0131533

**Published:** 2015-07-10

**Authors:** Davoud Torkamaneh, Francois Belzile

**Affiliations:** Département de Phytologie and Institut de Biologie Intégrative et des Systèmes (IBIS), Université Laval, Quebec City, QC, Canada; Agriculture and Agri-Food Canada, CANADA

## Abstract

Genotyping-by-sequencing (GBS) represents a highly cost-effective high-throughput genotyping approach. By nature, however, GBS is subject to generating sizeable amounts of missing data and these will need to be imputed for many downstream analyses. The extent to which such missing data can be tolerated in calling SNPs has not been explored widely. In this work, we first explore the use of imputation to fill in missing genotypes in GBS datasets. Importantly, we use whole genome resequencing data to assess the accuracy of the imputed data. Using a panel of 301 soybean accessions, we show that over 62,000 SNPs could be called when tolerating up to 80% missing data, a five-fold increase over the number called when tolerating up to 20% missing data. At all levels of missing data examined (between 20% and 80%), the resulting SNP datasets were of uniformly high accuracy (96–98%). We then used imputation to combine complementary SNP datasets derived from GBS and a SNP array (SoySNP50K). We thus produced an enhanced dataset of >100,000 SNPs and the genotypes at the previously untyped loci were again imputed with a high level of accuracy (95%). Of the >4,000,000 SNPs identified through resequencing 23 accessions (among the 301 used in the GBS analysis), 1.4 million tag SNPs were used as a reference to impute this large set of SNPs on the entire panel of 301 accessions. These previously untyped loci could be imputed with around 90% accuracy. Finally, we used the 100K SNP dataset (GBS + SoySNP50K) to perform a GWAS on seed oil content within this collection of soybean accessions. Both the number of significant marker-trait associations and the peak significance levels were improved considerably using this enhanced catalog of SNPs relative to a smaller catalog resulting from GBS alone at ≤20% missing data. Our results demonstrate that imputation can be used to fill in both missing genotypes and untyped loci with very high accuracy and that this leads to more powerful genetic analyses.

## Introduction

Next generation sequencing (NGS) has revolutionized plant and animal research in many ways. Firstly, it has allowed researchers to decode the whole genome of many organisms. Currently, hundreds of eukaryotic genomes have been sequenced (NCBI, “www.ncbi.nlm.nih.gov/projects/WGS/WGSprojectlist.cgi”) and, for some species, numerous individuals, cultivars or accessions of the same species have also been sequenced [1–3]. Next generation sequencing has also facilitated greatly the development of methods to genotype very large numbers of molecular markers such as single nucleotide polymorphisms (SNPs). In one such approach, large-scale sequencing has allowed researchers to probe nucleotide diversity in panels of individuals to discover polymorphic sites and then to develop genotyping arrays (“SNP chips”) that can subsequently be used to determine the genotype of an individual line at thousands to millions of such SNPs [4,5]. In soybean, an example of this approach is the SoySNP50K array that was constructed to interrogate over 52K SNPs of which 47,337 were found to be polymorphic among a set of 288 elite cultivars, landraces and wild soybean accessions [6]. Alternatively, genotyping methods exploiting the power of NGS technologies have also been developed to simultaneously identify and genotype SNPs. RAD-Seq (Restriction site Associated DNA Sequencing) and genotyping-by-sequencing (GBS) are two examples of such SNP genotyping approaches relying on NGS [7,8].

In soybean, GBS has been developed as a rapid and robust approach for reduced-representation sequencing of multiplexed samples that combines genome-wide molecular marker discovery and genotyping [9]. The flexibility and low cost of GBS makes this an excellent tool for many applications and research questions in genetics and breeding. Such modern advances allow for the genotyping of thousands of SNPs, and, in doing so, the probability of identifying SNPs correlated with traits of interest increases [10]. However, when using approaches such as GBS that perform a scan or a sampling of the genome, the quantity of missing data can be substantial. An important question that remains unanswered at this point is the degree to which missing data can be tolerated and to what extent they affect the accuracy of the imputation process.

Conceptually, there are two types of missing data in large datasets. The most obvious is when some individuals are missing a genotype value at a locus that is otherwise successfully typed in the other individuals of a population. In another situation, which arises when different datasets (e.g. obtained using different genotyping technologies) are combined, there can be loci that are not typed at all within a population, i.e. there is no information for a SNP locus in all individuals of the population except for a few individuals that can be common to both datasets. The first type of missing data can be termed a “missing genotype” while the second is termed an “untyped locus”. There has been considerable interest in imputing such missing data based on the available data [10]. Many tools used in genetic analysis require complete datasets and there are thus two possibilities: work only with SNP loci devoid of any missing data (thereby considerably reducing the number of SNPs available) or impute these missing data through various strategies.

Imputation is the substitution of some value for missing data, in other words, ‘filling in’ missing data with plausible values. Generally, methods of genotype imputation are based on the concept that SNPs close together on a chromosome are often inherited together. The resulting correlations among SNPs are referred to as linkage disequilibrium (LD), or association, in the genetic literature [11]. Many methods for imputing missing genotypes have been suggested and tested. Generally, two methodological classes are considered: regression and phasing.

A first approach is to use regression models to impute the missing genotypes by using flanking SNPs as covariates [10]. Regression-based methods face a common problem in variable selection; it can be difficult to select which available SNPs should be included as covariates. One reason for this is that LD patterns are not homogenous across the genome [11]; for example, lower LD would be expected among SNPs located in recombination hotspots than those in low recombination regions (high LD regions). Therefore, fewer SNPs may be useful as covariates in lower LD regions. These limitations made regression methods less attractive and less accurate.

Phase-based methods consider haplotype structure and common descent patterns [10]. As humans, animals, and plants are (often) diploid, a genotype is the combination of maternal and paternal alleles. Alleles close together on a chromosome are typically inherited together in a whole unit as a haplotype. Phase-based algorithms try to split genotypes at SNPs into haplotypic phases. Here, a “phase” is simply an inferred parental haplotype. Once phased, missing alleles can be estimated from neighboring haplotype alleles through their LD relationship, and the inferred alleles are then combined to impute the missing genotype. Currently, many popular genotype imputation methods are phase-based.

In this work, we explored the accuracy and efficiency of different imputation tools for both the imputation of missing genotypes in the context of GBS and of untyped loci in the context of combining SNP datasets obtained through different genotyping approaches (GBS, SNP array and resequencing). Finally, we examined the impact of using such enhanced SNP datasets in genome-wide association analyses.

## Materials and Methods

### Samples and SNP datasets

A set of 301 Canadian soybean lines was subjected to GBS analysis (with *Ape*KI digestion) and a total of 450 million 100-bp reads (~1.5M reads/line) were processed through our analytical pipeline that relies on SAMtools to call SNPs as described previously in Sonah et al. [9] and Sonah et al. [12]. The SoySNP50K iSelect BeadChip [6] has been used to genotype the USDA Soybean Germplasm Collection [13]. The complete dataset for 19,652 *G*. *max* and *G*. *soja* accessions genotyped with 42,508 SNPs are publicly available on Soybase (www.soybase.org). Of these 19,652 accessions, 25 were in common with the 301 Canadian lines used for GBS. Finally, on the basis of geographic distribution and genotypic diversity, we chose 23 soybean (S1 Table) lines from the set of 301 mentioned above to undergo whole genome resequencing (described below).

#### DNA extraction and whole genome resequencing

Seeds were planted in individual two-inch pots containing a single Jiffy peat pellet (Gérard Bourbeau & fils inc. Quebec, Canada). First trifoliate leaves from 12 day-old plants were harvested and immediately frozen in liquid nitrogen. Frozen leaf tissue was ground using a Qiagen TissueLyser. DNA was extracted from approximately 100 mg of ground tissue using the Qiagen Plant DNeasy Mini Kit according to the manufacturer’s protocol. DNA was quantified on a NanoDrop spectrophotometer. Illumina Paired-End libraries were constructed for DNA samples using the Illumina Tru-seq DNA Library Prep Kit (Illumina, San Diego CA, USA) following the manufacturer’s instructions. DNA library quality was verified on an Agilent Bioanalyzer with a High Sensitivity DNA chip. Samples were sequenced using the Illumina HiSeq 2000 platform at the McGill University-Génome Québec Innovation Center in Montreal, QC, Canada.

#### Alignment and variant calling

Illumina paired-end reads were aligned using the Burrows-Wheeler Aligner (BWA) [14] onto the soybean reference genome (Williams82) [15]. Variants were called using SAMtools 0.1.18 [16]. BAM files were pooled for variant calling. Variants were then removed if they had two or more alternative alleles, no observation of the alternative allele on either forward or reverse reads, an overall quality (QUAL) score of <20, a mapping quality (MQ) score <30, a read depth of <2, or were suspected of representing false heterozygotes (based on unequal read depth of the two alleles). For tag SNP selection, we used PLINK [17] to calculate linkage disequilibrium (LD) between each pair of SNPs within a sliding window of 50 SNPs and we removed all but one SNP that were in perfect LD (LD = 1); the remaining SNPs were deemed tag SNPs.

#### Imputation methods

We used three software tools to impute missing data: fastPHASE [18], BEAGLE v4.0 [19], and IMPUTE2 [20]. As recommended by Delaneau, and Marchini [21] we used SHAPEIT2 [22] to first infer the haplotypes among the set of genotypes studied, and then used the resulting output to perform the imputation of untyped loci using IMPUTE2. All three software tools were used to impute missing genotypes while only the last two were used to impute untyped loci. The parameters for fastPHASE were: fastPHASE –T 20 –E 10 –M 0 –o output_name fastPHASE_input_file. The command line for BEAGLE read as follows for missing data imputation: java—Xmx5000m —jar unphased = phased.input.bgl missing = 0 niterations = 10 out = out_file, and for untyped genotype imputation: java—Xmx5000m —jar phased = phased.input.bgl unphased = unphased.input.bgl markers = marker.ids missing = 0 niterations = 10 out = out_file. Finally, the command line for IMPUTE2 was: impute –h phased_file—l legend_file—g geno_file –m genetic_map_chr*.txt—call_-thresh 0.0—Ne 11418—i info_file –o out_file. Finally, both BEAGLE and IMPUTE2 were used to assess the impact of the number of lines composing the reference panel on the accuracy of imputation at untyped loci.

#### Genotype accuracy

For the initial estimation of the accuracy of genotype calls in GBS analysis, we compared the called genotypes at all loci on a single chromosome (Gm03; 3326 SNP loci) for the 23 lines common to both the GBS and WGS datasets. These GBS-derived genotypes were directly compared with the true genotypes (revealed by WGS) using an in-house script. Similarly, all imputed genotype calls (initially missing data) on Gm03, following imputation (three imputation methods, as described above, at the different levels of MaxMD and MinMAF), were compared with the true genotypes (WGS). To verify that this chromosome was representative of the broader genome, we estimated the overall genotype accuracy (GBS-derived and imputed SNPs) for all chromosomes (Gm01 to Gm20) using BEAGLE only and at MaxMD≤80% and MinMAF = 0.003.

To assess the accuracy of imputation at untyped loci when combining GBS and SoySNP50K datasets, i.e. when the SoySNP50K data were used as a reference panel to impute genotypes at loci not common to both datasets we extracted the genotypes at all loci on chromosome Gm03 for three lines (Maple Presto, Mandarin, and Evans) for which WGS, GBS, and SoySNP50K data were available. Imputed SNP genotypes were compared with the true genotypes revealed by WGS.

Similarly, to assess the accuracy of imputation at untyped loci that were imputed using the WGS dataset, we used the WGS SNP data from 22 of the 23 resequenced lines as a reference panel to impute these SNPs onto the GBS or GBS + SoySNP50K data. The remaining line was kept for validation of the imputed SNPs. We performed three permutations where a single line was kept aside to estimate imputation accuracy (S3 Table). We then extracted the genotypes at all loci on chromosome Gm03 for the remaining line and we directly compared with the true genotypes.

#### Genome-wide association study

A subset of 139 soybean lines were used in the GWAS analysis. Phenotypic data (seed oil content) for these lines were originally described by Sonah et al. [12]. All the analyses were performed using the Genomic Association and Prediction Integrated Tool (GAPIT) [23]. A general linear model (GLM) was used with or without the covariate P from principal component analysis (PCA) and a kinship matrix was calculated either using the VanRaden method (K) or the EMMA method (K*) to determine relatedness among individuals [23]. A multi-locus mixed model (MLMM) incorporating a kinship matrix (K or K*) along with a P or Q matrix was used to test for marker-trait association [24]. The negative log(1/*p*) was used to establish a significance threshold [25, 26].

## Results

### Factors that affect number of SNPs in GBS analysis

**Fig 1:**
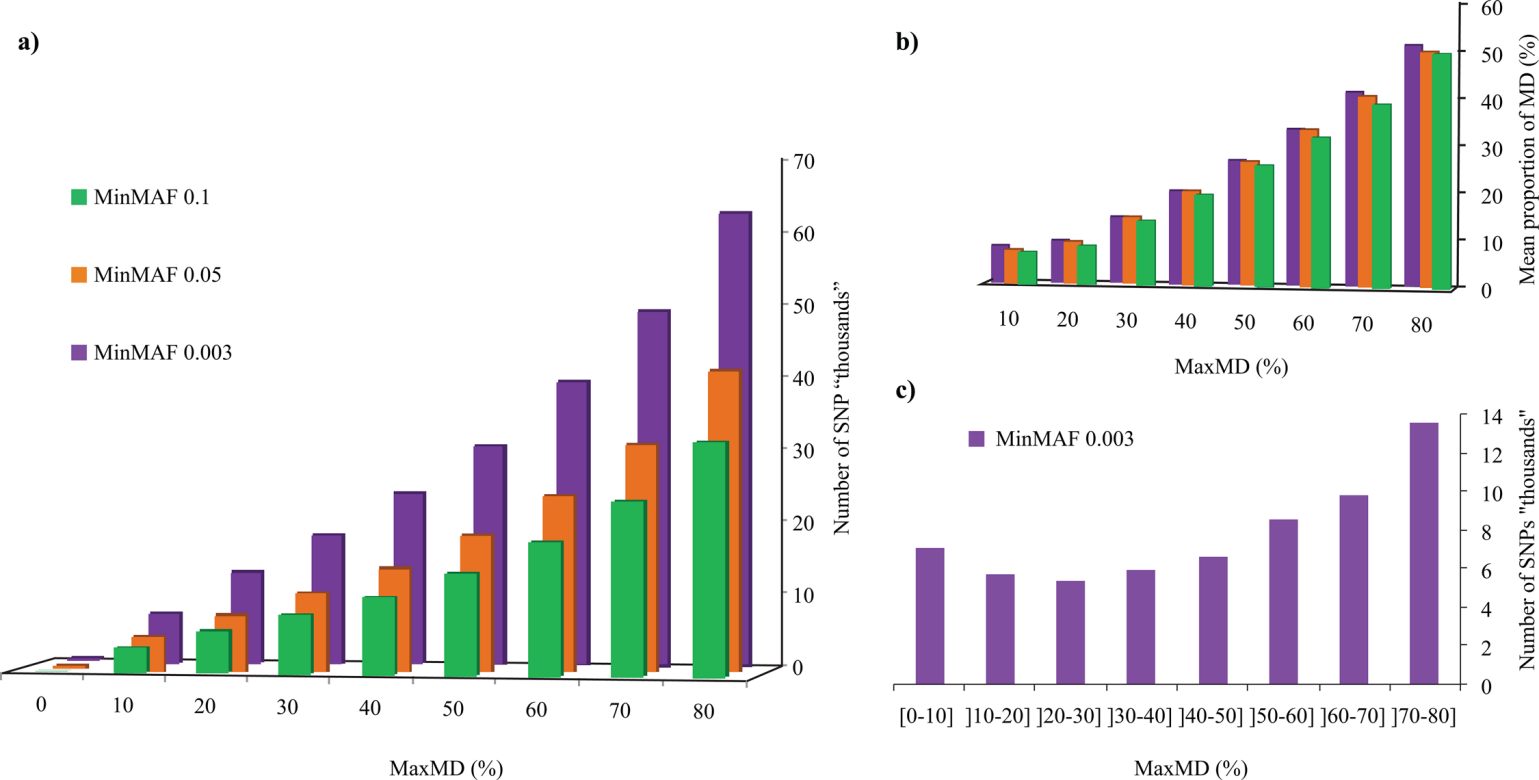
Impact of missing data and minor allele frequency on the number of SNPs. (a) The number of SNPs (in ‘000’s) is plotted as a function of the maximal proportion (in %) of missing data tolerated (MaxMD) at three levels of minimal minor allele frequency (MinMAF). (b) Overall mean proportion of missing data (in %) for datasets obtained at different levels of MaxMD and MinMAF. (c) Distribution of SNPs called at different levels of missing data.

We first explored the impact of two key filtering steps central to the production of SNP catalogs derived from GBS analysis: the maximal amount of missing data allowed (MaxMD, in %) and the minimal minor allele frequency (MinMAF). A set of 301 Canadian soybean lines was subjected to GBS analysis and a total of 450 million 100-bp reads (mean of ~1.5M reads/line) were processed through our analytical pipeline that calls SNPs using Samtools (see materials and methods for details). Using a minimum of one read to call a genotype, we obtained an initial catalog of 247,851 SNPs. We then filtered this set of SNPs for both MaxMD (between 0 and ≤80% missing data) and for MinMAF (0.003, 0.05 and 0.1). As can be seen in Fig 1A, the amount of missing data allowed had a very large impact on the number of SNPs retained. At a MinMAF of 0.003 (i.e. a single line carrying a different allele among 301 lines), the number of SNPs increased steadily from only 1 (0% missing data) up to 62,643 (≤80% missing data). At the other MinMAF values, SNP numbers similarly increased markedly between 0 and 41,024 (MinMAF = 0.05) and between 0 and 32,035 (MinMAF = 0.1).

As the MaxMD filter only reflects the maximal proportion of missing data that are tolerated for an individual SNP marker to be retained, it does not accurately reflect the actual mean amount of missing data that characterizes a SNP dataset. To better capture this, we plotted the mean proportion of missing data at each of the MaxMD and MinMAF levels described above (Fig 1B). As can be seen, the proportion of missing data in an entire dataset was hardly affected by the MinMAF threshold used but was heavily impacted by the chosen MaxMD level. Even at MaxMD of 80%, the mean amount of missing data was around 50%, while at more stringent MaxMD levels (e.g. 20%), the mean proportion of missing data became quite low (<10%).

We then examined the distribution of these SNPs based on the amount of missing data (in successive increments of 10%) at the most permissive MinMAF level (0.003). As can be seen in Fig 1C, over 13,000 SNPs were called with >70% and ≤80% missing data, while around 7,000 were called with ≤10% missing data. Globally, approximately half of the SNPs could be called with ≤50% missing data while the other half were called with between 50% and 80% missing data. We therefore conclude that it is possible to quite significantly increase the number of called SNPs by allowing for more missing data, but this will only be attractive if these missing data can be accurately imputed.

### Accuracy and efficacy of imputation for missing genotypes

To examine the quality of the SNP data obtained using GBS, we first assessed the accuracy of the SNP genotypes initially called by GBS, prior to any imputation. To achieve this, we performed whole-genome resequencing on a representative subset of 23 soybean lines at a mean depth of coverage of 9x (genome coverage of 96%) (S1 Table). A total of 3.6M SNPs were called among these lines and this dataset was presumed to represent the true genotype at variant positions. Assessments of the accuracy of called or imputed SNPs were performed on SNPs located on a single chromosome (Gm03) for all methods at different levels of MaxMD and MinMAF. At a MaxMD of 80% and MinMAF of 0.003, we found that 98.4% of SNP genotypes called by our GBS pipeline proved to be identical to the true genotypes. Similar levels of accuracy were found for called SNPs under all filtering conditions (data not shown).

**Fig 2:**
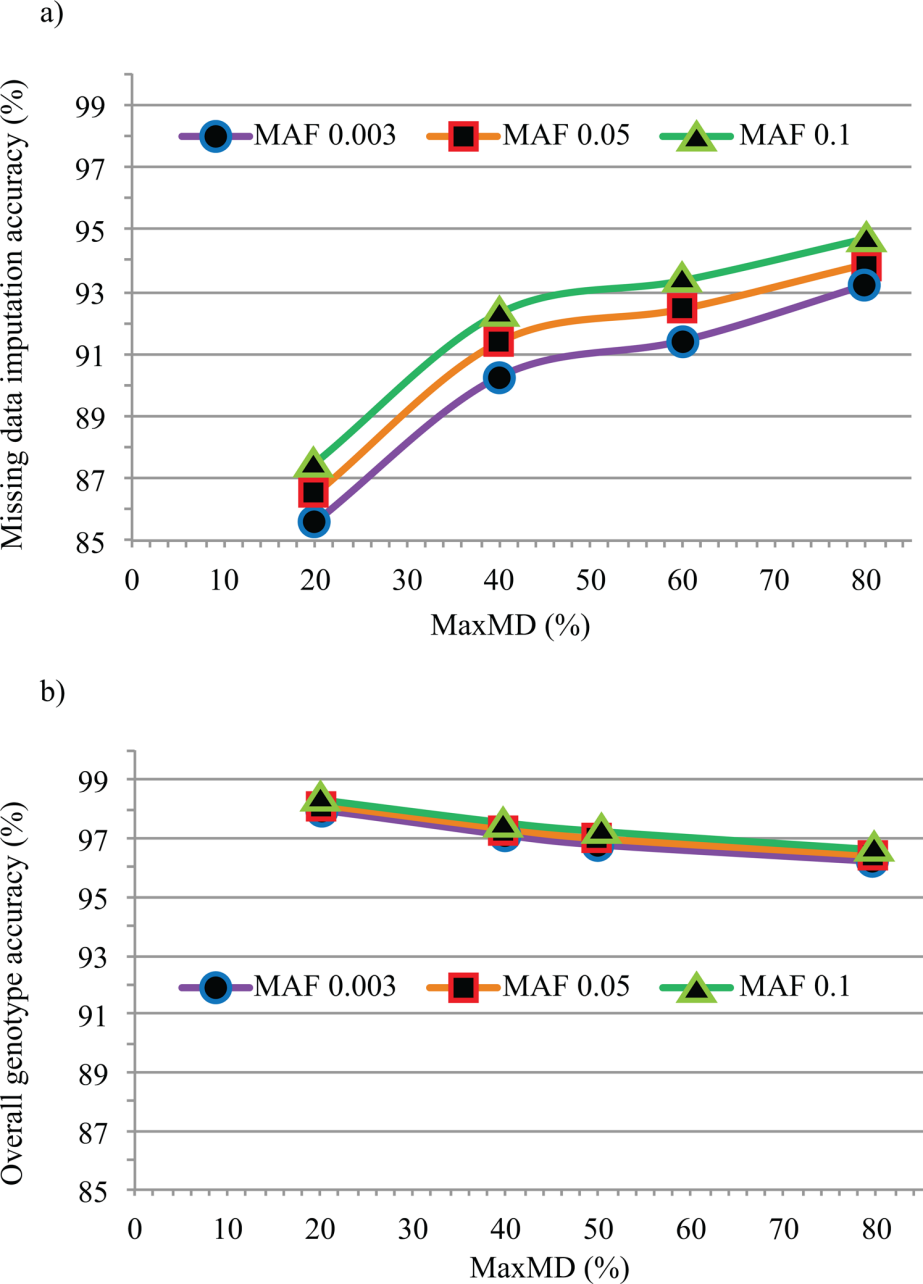
Missing data imputation accuracy. (a) The accuracy of imputed missing data (in %) is plotted against the proportion of missing data (in %) tolerated (MaxMD) at three levels of minimal minor allele frequency (MinMAF). (b) Accuracy of overall GBS dataset (in %) after imputation at different levels of MaxMD and MinMAF.

In a second step, to estimate the accuracy of imputed SNP data (i.e. formerly missing genotypes), we performed imputation at all levels of MaxMD and MinMAF on the entire set of 301 lines. Once again, we used the resequencing data as a reference and, as shown in Fig 2A and detailed in Table 1, we found that imputation accuracy was hardly affected by the chosen minor allele frequency and only moderately affected by the amount of missing data. Somewhat surprisingly, the accuracy of imputation actually increased with increasing missing data. Indeed, while the imputation accuracy was 86% at MaxMD = 20%, it rose steadily to reach 94% at MaxMD = 80%. Therefore, allowing for a greater amount of missing data not only yielded a larger number of SNP markers, but this also proved beneficial in terms of the accuracy of imputed genotypes.

**Table 1:** Accuracy of imputed GBS SNP data and computational speed of three imputation methods at different levels of missing data (MaxMD) and minor allele frequency (MinMAF).

			Missing data imputation accuracy (%)						
			MinMAF 0.003		MinMAF 0.05		MinMAF 0.1		
Method	Dataset	MaxMD (%)	Missing data	Overall*	Missing data	Overall	Missing data	Overall	Computing Time
									
**fastPHASE**	GBS**	80	93.2	95.8	93.9	96.4	94.1	96.5	14 hours
** **		20	85.6	97.5	86.5	98.1	87.5	98.1	
** **									
**BEAGLE**	GBS	80	92.9	95.6	94.0	96.5	94.2	96.6	30 minutes
** **		20	85.6	97.5	86.7	98.1	87.6	98.1	
** **									
**IMPUTE2**	GBS	80	93.0	95.6	93.5	96.2	94.3	96.6	2 hours
** **		20	86.1	97.5	86.9	98.1	88.1	98.2	
									
**Number of SNPs**	GBS	80	62,643		41,024		32,035		
** **		20	12,712		7,152		5,657		

* Includes both genotypes originally called by GBS and following imputation

** 301soybean lines

As illustrated above, the proportions of called and imputed SNP genotypes did vary at different MaxMD levels and thus impacted the overall accuracy of the resulting SNP catalog. The accuracy of the entire GBS-derived SNP dataset (after imputation) was measured and is illustrated in Fig 2B and detailed in Table 1. This includes both the SNP genotypes initially called and those resulting from imputation. Overall genotype accuracy ranged between 96% (MaxMD = 80%) and 98% (MaxMD = 20%), with hardly any impact of the MinMAF level. To determine if Gm03 was representative of the entire set of 20 chromosomes, we measured overall genotype accuracy for all chromosomes using a single imputation tool (BEAGLE) at a single level of MaxMD and MinMAF (80% and 0.003, respectively). As shown in S2 Table, imputation accuracy differed very little between chromosomes, ranging between 95.3% and 96.3% (mean = 95.84% ± 0.28%).

Finally, although all three software tools performed equally well in terms of accuracy of imputation, computational speed varied considerably (Table 1). Whereas it took fastPHASE 14h to impute the missing data, BEAGLE completed the task in only 30 minutes. In conclusion, we find that large amounts of missing data do not have a significant detrimental impact on the overall accuracy thanks to a highly accurate imputation.

### Accuracy of imputation at untyped loci

The existence of multiple genotyping approaches offers the opportunity to exploit already existing haplotype information to further enhance marker density and to facilitate the integration of data obtained from different genotyping platforms. We first wanted to test whether the publicly available SoySNP50K array data obtained on 19,562 USDA soybean accessions could be used to impute additional (“untyped”) SNPs in our GBS-derived catalog of SNPs. In a first step, we identified 25 accessions common to our set of 301 lines and the USDA collection. By comparing the SNP data for these common accessions, we found that only 7% of markers (2,975 of 42,508; at MAF = 0.05) were shared between the GBS and SoySNP50K data. As these two datasets have a limited overlap, this offered the potential of adding a large number of untyped SNP loci through imputation. In a second step, we used the SoySNP50K data as a reference panel to perform imputation of genotypes at the untyped loci in our GBS-derived catalog. As shown in Table 2, both BEAGLE and IMPUTE2 performed very well resulting in a high accuracy of the imputed genotypes (94.9 and 95.3%, respectively). The successful imputation of these untyped loci increased the number of SNP markers from 62,643 to 102,175, all the while maintaining a high level of accuracy of the combined catalog of SNPs.

**Table 2:** Accuracy and computational efficiency of imputation at untyped loci. SNP data from a SNP array (SoySNP50K) or whole-genome resequencing (WGS) were used as a reference to impute missing data at loci that were untyped in an initial dataset (GBS data only or GBS +SoySNP50K data).

Dataset	Imputation method	Reference panel	Untyped loci imputation accuracy (%)	Number of markers	Computing Time
	**BEAGLE**				
GBS	Beagle	SoySNP50K	94.9	102,175	71 hours
GBS	Beagle	WGS	80.0	1,414,925	2 hours
GBS+ SoySNP50K	Beagle	WGS	88.1	1,312,760	2 hours
	**IMPUTE2**				
GBS	pre-Phasing by SHAPIT2	SoySNP50K	95.3	102,175	91 hours
GBS	pre-Phasing by SHAPIT2	WGS	90.0	1,414,925	7 hours
GBS+ SoySNP50K	pre-Phasing by SHAPIT2	WGS	91.8	1,312,760	8 hours

Another source of haplotype information resided in our WGS data on the subset of 23 resequenced Canadian lines. We therefore tested how useful this information could be in terms of imputing an even larger set of untyped loci. As described above, a total of 3.6M SNPs were identified among these 23 lines. We removed all redundant markers, i.e. SNPs that were in perfect LD with at least one other SNP, thus reducing this reference panel to 1.4M tag SNPs. We then used BEAGLE and IMPUTE2 for imputation using the SNP data from 22 lines as a reference panel and keeping the last line (Gaillard) for the estimation of accuracy. As shown in Table 2, the accuracy of imputed genotypes ranged from as low as 88% to as high as 91.8%. Again, differences in computation time were observed with BEAGLE proving to be the most efficient.

Finally, to ensure that these results were broadly applicable to the larger set of 23 lines, two additional permutations were done where a different set of 22 lines was used as a reference panel and the remaining line (Mandarin or OAC-Lakeview) used for validation. Here again, the accuracy of imputation proved highly similar to the results described above, ranging between 87.9% and 92.4% (S3 Table).

**Fig 3:**
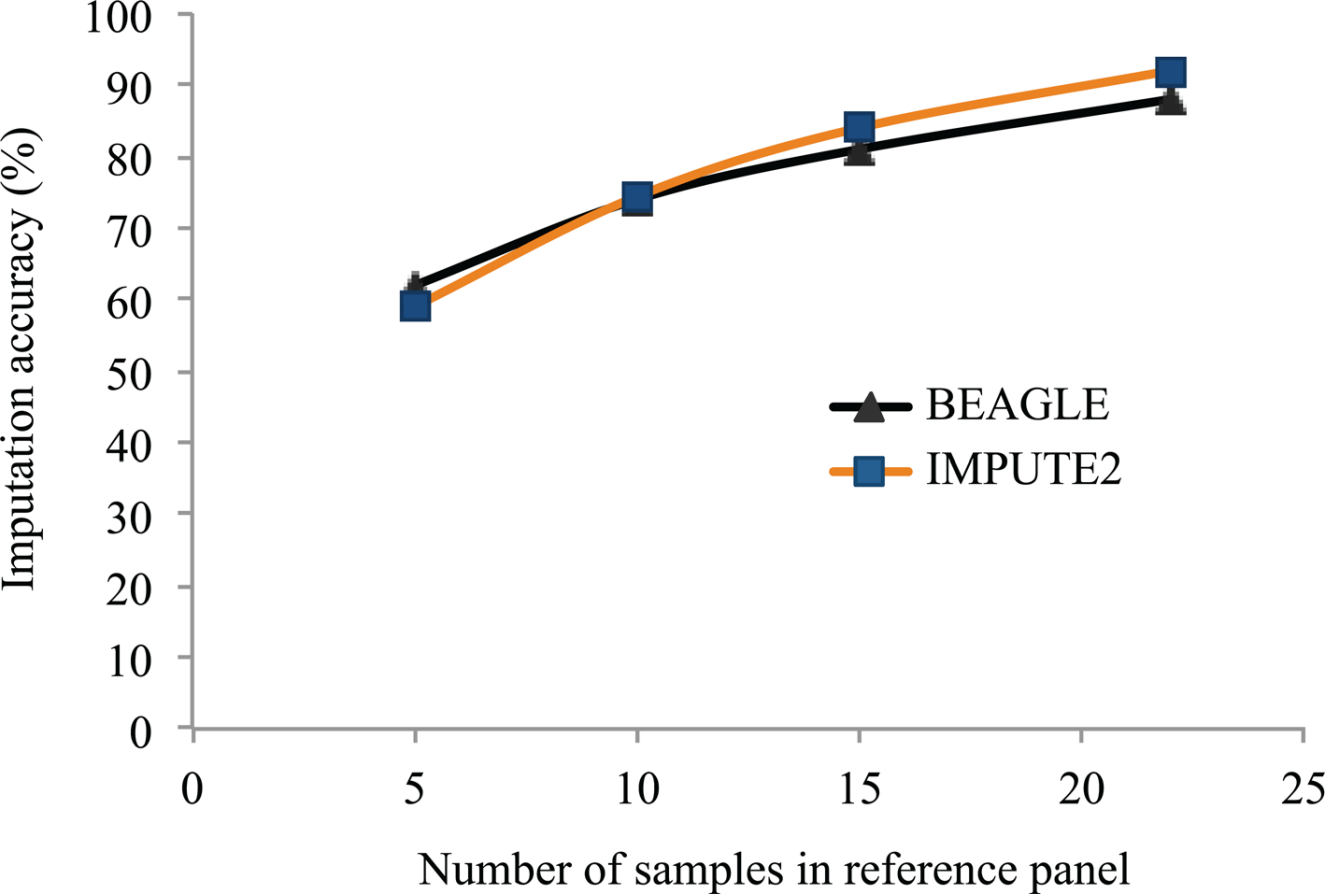
Imputation accuracy at untyped SNPs using reference panels of different sizes. SNPs identified through resequencing of a varying number (5 to 22) soybean accessions were used as a reference panel to impute the genotypes at these SNP loci in a set of 301 soybean accessions using two different imputation softwares (BEAGLE and Impute2).

To explore the impact of the size of this reference panel on the accuracy of imputed SNPs, we performed imputation with reference panels representing subsets of 5, 10, 15 or 22 of the 23 lines for which WGS data were available. As can be seen in Fig 3, the accuracy of imputation was highly affected by the number of lines used in the reference panel. With only 5 lines included in the reference panel, imputation accuracy was low (60% with BEAGLE and 59% with IMPUTE2) while it increased (to 88% with BEAGLE and 91.8% with IMPUTE2) using the maximum number of lines available (22). This suggests that a further increase in the number of lines included in the reference panel could provide an increase in the accuracy of the imputation of untyped loci.

### Power of association test using imputed data

**Fig 4:**
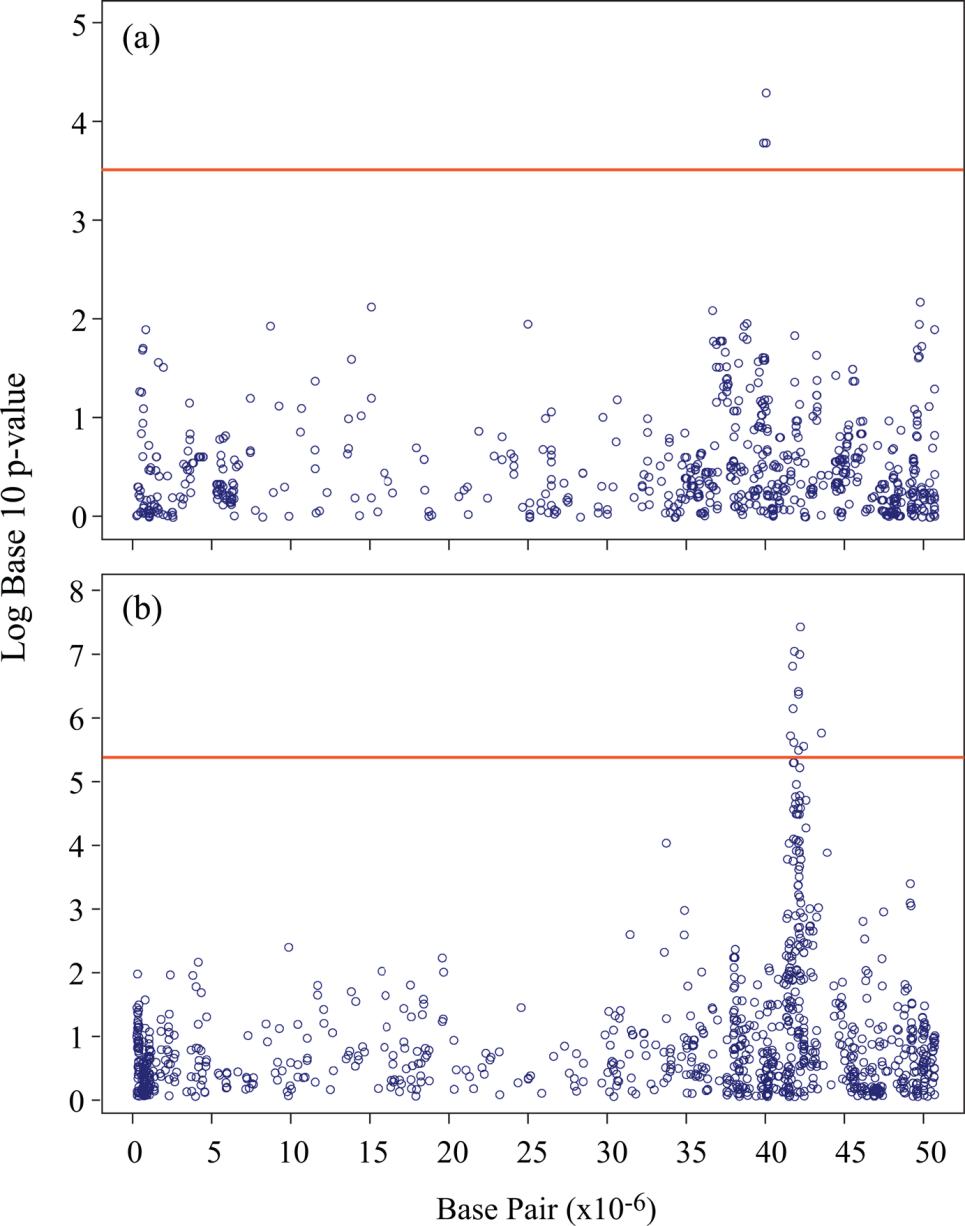
Association analysis for seed oil content on chromosome 19 (Gm19) in soybean. Negative log10 *p*-values from a genome-wide scan are plotted against marker positions on chromosome 19. (a) Association analysis with the original GBS dataset (~7K SNPs). (b) Association analysis with the enhanced SNP dataset (>83K SNPs) after combining GBS and SoySNP50K data via imputation.

To determine if the enhanced SNP catalogs obtained through imputation could provide increased power in genome-wide association scans, a subset composed of 139 soybean lines was used to perform an association analysis for seed oil content. This subset was used because phenotypic data were available only for these lines. One analysis was conducted using a “basic” GBS catalog of 7,152 SNPs obtained at MaxMD = 20% and MinMAF = 0.05, while the other was performed using an enhanced catalog resulting from imputation of missing GBS data (at MaxMD = 80%) and untyped loci from the SoySNP50K dataset. At MAF≥0.05, a total of 83,532 SNPs were retained within this combined dataset. As can be seen in Fig 4A, using the “basic” SNP catalog, a single SNP marker on Gm19 showed a significant association (*p* = 9.6×10^−3^ and *q* = 0.09) with seed oil content. In contrast, using the enhanced SNP catalog (Fig 4B) and a multi-locus mixed-model implementation, a total of 11 markers were in significant association with this trait despite the fact that the significance threshold increased from 3.4 to 5.3 (-Log_10_ *p-*value). Interestingly, the peak SNP in both cases was the same (Gm19_41742182), but its association with oil content exhibited a much higher *p*-value (3.1×10^−7^) and lower *q*-value (0.01). This demonstrates that the increased number of informative SNP loci, obtained through the imputation of both missing GBS data and untyped loci from additional sources of SNP haplotype information, can prove highly beneficial in studying the genetic architecture of complex traits.

## Discussion

A first key element to come out of this work is that MaxMD is the most important factor determining the number of SNPs in GBS analysis. As seen in this study when increasing MaxMD from 20 to 80% incrementally, the number of SNPs called increased from 12,712 to 62,643. As previously described, one of the unique features associated with GBS is the generation of highly incomplete SNP genotype data [27–30], largely due to low coverage sequencing [7]. The incompleteness could be up to 90% of observations missing [27,31]. As described in several GBS studies in different species (maize, rice, wheat, soybean, and barley), increasing the amount of missing data allows to capture more SNPs [32–36]. In the most closely related work, Jarquin et al. [35] observed a 4-fold increase in the number of SNPs scored in elite soybean breeding lines when increasing the percentage of missing data allowed from 5% to 80%. These data confirm that with increasing MaxMD the number of SNPs called through GBS can be increased substantially.

As described, the number of SNPs is also affected by MinMAF, but the overall proportion of missing data is hardly affected. The effect of MinMAF on the number of SNPs has been described in several reports. The number of SNP increases as the minor allele frequency decreases [34,35,37]. These authors, however, did not show the relation between MinMAF and the proportion of missing data. In this study, we demonstrated that the proportion of missing data is largely independent of the chosen MinMAF. In a practical context, however, there is a more limited scope for using a broad range of MinMAF values, as these are usually constrained by the need to have an adequate representation of the minor allele state. Typically, in GWAS and other similar genetic studies, the most frequently encountered MinMAF values are 0.05 and 0.10. In contrast, the amount of missing data that is tolerated is much more variable across studies and is mostly constrained by the quality of the imputation that can be achieved when filling in these missing data.

Somewhat counterintuitively, a second key result of this work was that imputation of missing data was more accurate when performed on datasets with a higher proportion of missing data. Indeed, at MaxMD = 80%, 94% of SNP genotypes were correctly imputed, whereas at MaxMD = 20%, the accuracy decreased to 86%. Upon reflection, however, it seems logical that a larger number of SNP markers (albeit with more missing data) better captures the diversity of haplotypes that are present within a collection of lines. Increased imputation accuracy at MaxMD = 80% is likely achieved through increased LD between markers. As documented by Zheng et al. [38], imputation accuracy increases with increasing density of markers. Soybean has high levels of LD and the average distance over which LD decays to half of its maximum value in soybean is substantially longer than that of many plants and animals analyzed to date (cultivated soybean: ~150 kb; wild soybean: ~75 kb; cultivated rice: <65–180; wild rice <10 kb; maize: <1 kb; and *Arabidopsis thaliana*:~3–4 kb; humans <5kb; cattle <10kb) [39–44]. High levels of LD will decrease the haplotype diversity and as a result facilitate the imputation of missing data even over long distances. This suggests that imputation accuracy will vary with differing levels of LD in different species.

A novel aspect of this work is that the measurement of the accuracy of imputation was assessed by comparing directly to whole genome resequencing data obtained for a subset of the lines. In many previous studies, estimates of the accuracy of imputation have been achieved by masking a subset of the data, imputing these missing genotypes, and then comparing the imputed genotype with the original data [32,35–37]. For the most part, similarly high levels of imputation accuracy (92–98%) have been reported with slight differences being observed between species and types of population (related or unrelated individuals). The advantage of using resequencing data in this fashion is that we can assess the accuracy of imputation at a specific level of missing data without having to add to this by masking a subset of the available data.

Furthermore, although the threshold for retaining a SNP marker at MaxMD = 80% would suggest a tremendous amount of missing data, we showed that, averaged across all markers kept at this threshold, a mean of 50% missing data was obtained. When we considered jointly both the called and imputed markers comprising the final dataset at the various missing data levels, all were highly accurate (96–98%). This is because the genotypes initially called via GBS analysis are themselves highly accurate (98.4%). At MaxMD = 20%, these high-quality SNPs are combined with a small proportion (7%) of SNPs imputed with what we term a “good” accuracy (84%). At the other end of the missing data spectrum (MaxMD = 80%), the original set of GBS-called SNPs is combined with an equal amount (~50%) of SNPs derived from imputation with an only slightly lower accuracy (94%). Thus, catalogs of called and imputed SNPs retain a constant, high level of accuracy (~97%) across a broad range of missing data thresholds.

A third key finding of this work is that different and highly complementary marker datasets can be successfully combined via imputation at untyped loci. We showed that SNP catalogs derived from two high-throughput genotyping techniques, GBS and a SNP array (SoySNP50K), could be fused through the imputation of a large number of untyped loci. Because of the different composition of the two initial catalogs, only 7% of the GBS markers were present in the SoySNP50K set. This is because most (90%) of the SoySNP50K markers are present in genic regions [6], while most of the GBS markers are present in intergenic regions (29.8%) or downstream regions (20.2%) [9]. We nonetheless successfully imputed ~40K SNPs from the array that were absent from the GBS dataset with a high level of accuracy (95%). By doing so, our catalog of SNPs for the collection of 301 Canadian soybean lines was enhanced and exceeded 100K SNPs. This analysis shows that GBS and SNP arrays are highly complementary approaches that can be used in parallel and combined. As the SoySNP50K has been used by the USDA to characterize close to 20,000 lines of soybean, and because these data are public, any researcher anywhere in the world can make use of this data, in combination with their own GBS-derived data obtained at a very low cost, to achieve excellent genome coverage. Similarly, Pei et al. [45] and Hao et al. [46] used imputation to combine data from two human genotyping arrays: the Affymetrix 500k SNP chip and the Illumina 550k chip with HapMap SNPs. They showed that the accuracy of imputation at such untyped loci using various tools (BEAGLE, fastPHASE, and IPMUTE2) ranged between 92 and 94%. We suggest that the higher level of imputation accuracy observed in this study compared to the human dataset is because of the high level of LD in soybean. Again this result suggests that the accuracy of genotype imputation at untyped loci will vary in different species because of stark differences in the extent of LD. Overall, a competing genotyping platforms are developed, it is good to know that researchers can produce high-quality integrated data sets offering better genome coverage by such imputation of untyped loci.

Although all imputation softwares use the same fundamental phenomenon of LD across the genome, the algorithms employed by each package differ. Likewise, each package offers differing strengths and weaknesses. Therefore, it is a good idea to use more than one software package, compare results, and investigate any major discrepancies [47]. To perform genotype imputation, we used three imputation softwares and found that these showed approximately the same level of accuracy for missing data imputation. In our view, BEAGLE proved the most attractive, as it ran very quickly and was the most user friendly. As reference panels for the imputation of untyped loci become larger and larger, thanks to the increasing availability of data derived from the resequencing of an increasing number of soybean lines, these tools will gain further utility. In the context of this work, genotype imputation using the SoySNP50K data as a reference, both BEAGLE and IMPUTE2 showed the same accuracy (95%). Contrary to most previous work, we did not assess the accuracy of our imputation through the masking of a subset of available data. Rather, we performed whole-genome resequencing of a subset (23 lines) from our study population (301 lines) and we compared directly the imputed genotype and the true genotype. This analysis showed the high level of imputation accuracy.

When performing imputation at a much larger scale, using the 1.4M tag SNPs identified in our resequencing effort, the accuracy of imputation of this large number of untyped loci was dependent on the number of lines included in the reference panel. When increasing the number of lines composing the reference panel from only 5 to a maximum of 22, imputation accuracy increased from ~60% to close to 90%. Similarly, in humans, Li et al. [10] showed that increasing the number of individuals in the reference panel from 60 to 500 improved the accuracy of imputation (from 85% to more than 95%, respectively). Interestingly, even a small number of soybean lines (22) resulted in higher imputation accuracy than was achieved with 60 human samples. As LD is much more extensive in soybean than in humans, this again illustrates how important this factor will be in determining imputation accuracy. In future, to achieve a level of accuracy similar to that seen using the SoySNP50K data (95%), more lines from the Canadian germplasm collection would likely need to be sequenced.

A final key finding of this work is that the much increased marker coverage achieved through a better exploitation of available GBS and SoySNP50K data is highly useful in the genetic dissection of complex traits. The availability of higher density marker coverage enables researchers to more accurately determine which regions to investigate further and actually narrow down each region on which they should perform fine mapping. As illustrated in our analysis of seed oil content, the use of an enhanced SNP catalog (~6 fold larger) allowed us to capture more significant marker-trait associations around candidate QTLs and the significance level of such associations was also much higher. These results are consistent with recent work in both animals and plants that have demonstrated the benefits of marker imputation for GWAS [48,49]. In the latter case, the authors compared the benefits of marker imputation on the accuracy of measures of relatedness, the accuracy of genomic selection and the power to detect QTLs through GWAS. In this work, these authors concluded that “association mapping profited most from imputing missing values”.

As seen in this study, genotype imputation represents an essential tool in the analysis of high-throughput genotypic data. One of the most common criticisms regarding GBS is the presence of a substantial amount of missing data. Our data show that this can largely be overcome in soybean thanks to highly accurate imputation of missing genotypes. Furthermore, genotype imputation is particularly useful for combining results across studies that rely on different genotyping platforms. As different groups may use different genotyping tools, it is highly important to be able to produce integrated datasets that include all such markers to facilitate the exchange of knowledge and information. It is important to remember, however, that imputation accuracy will be affected by the extent of LD in the population/species studied. Finally, a further benefit of such imputation is that it increases the power of individual scans thanks to more extensive marker coverage. In the coming years, we expect these imputation-based analyses will become a key tool in the analysis of massively parallel shotgun sequence data enabling geneticists to rapidly deploy these technologies to analyze large samples and dissect the genetic basis of complex traits.

## Supporting Information

S1 Table List of resequenced samples with the number of reads and bases. (DOCX)

S2 Table Overall accuracy of genotypic data following GBS analysis and imputation of missing data for all 20 soybean chromosomes. Missing data were imputed using BEAGLE at MaxMD = 80% and MinMAF = 0.003.
(DOCX)

S3 Table Imputation accuracy of genotypes at untyped loci using whole-genome sequence data as a reference panel. Genotype data (1.4M SNPs) for 22 resequenced soybean lines were used to impute genotypes at loci that were untyped in the GBS SNP dataset while a 23rd resequenced line (either Gaillard, Mandarin or OAC-Lakeview) was set aside for validation. The imputed genotypes were compared to the result of the resequencing in the validation line. Three different permutations of the data (each time leaving out a single resequenced line from the reference panel) were performed.
(DOCX)
