# Supplementary material for: Scanning and Filling: Ultra-Dense SNP Genotyping Combining Genotyping-By-Sequencing, SNP Array and Whole-Genome Resequencing Data
Source: PLoS One. 2015 Jul 10;10(7):e0131533. doi: 10.1371/journal.pone.0131533 (PMC4498655; doi:10.1371/journal.pone.0131533)
Supplement: S1 Table — (DOCX) [file pone.0131533.s001.docx]

**S1 Table. List of resequenced samples with the number of reads and bases.**

| Sample | Number of Reads (PE*) | Number of Bases |
| --- | --- | --- |
| OAC-Orford | 50 401 243 | 10 080 248 600 |
| Gaillard | 72 779 030 | 14 555 806 000 |
| OAC-Bayfield | 54 476 049 | 10 895 209 800 |
| Maple_Glen | 40 443 051 | 8 088 610 200 |
| OT94-47 | 36 019 874 | 7 203 974 800 |
| OAC-Champion | 40 955 178 | 8 191 035 600 |
| Dares | 43 857 912 | 8 771 582 400 |
| OAC-Lakeview | 46 501 650 | 9 300 330 000 |
| OAC-Morris | 42 193 094 | 8 438 618 800 |
| OAC-Thames | 36 279 267 | 7 255 853 400 |
| Alta | 44 399 003 | 8 879 800 600 |
| AC-Proteina | 52 144 192 | 10 428 838 400 |
| OAC-Wallace | 41 982 923 | 8 396 584 600 |
| Mandarin | 49 771 834 | 9 954 366 800 |
| OAC-Kent | 49 397 571 | 9 879 514 200 |
| OAC-Ayton | 40 549 216 | 8 109 843 200 |
| Maple_Presto | 39 684 574 | 7 936 914 800 |
| Prudence | 36 410 197 | 7 282 039 400 |
| Prodigy | 42 822 685 | 8 564 537 000 |
| Evans | 41 387 675 | 8 277 535 000 |
| OAC-Erin | 41 267 871 | 8 253 574 200 |
| Majesta | 41 111 261 | 8 222 252 200 |
| OAC-Petrel | 44 288 503 | 8 857 700 600 |

* PE: Paired-ends
