# Supplementary material for: Scanning and Filling: Ultra-Dense SNP Genotyping Combining Genotyping-By-Sequencing, SNP Array and Whole-Genome Resequencing Data
Source: PLoS One. 2015 Jul 10;10(7):e0131533. doi: 10.1371/journal.pone.0131533 (PMC4498655; doi:10.1371/journal.pone.0131533)
Supplement: S2 Table — Missing data were imputed using BEAGLE at MaxMD = 80% and MinMAF = 0.003. (DOCX) [file pone.0131533.s002.docx]

| Chromosome | Length (bp) | Number of SNPs | Overall accuracy (%) |
| --- | --- | --- | --- |
| Gm01 | 55 915 595 | 2 608 | 96.2 |
| Gm02 | 51 656 713 | 3 339 | 95.8 |
| Gm03 | 47 781 76 | 3 326 | 95.6 |
| Gm04 | 49 243 852 | 3 929 | 95.6 |
| Gm05 | 41 936 504 | 2 390 | 95.4 |
| Gm06 | 50 722 821 | 3 301 | 95.8 |
| Gm07 | 44 683 157 | 2 654 | 95.6 |
| Gm08 | 46 995 532 | 2 827 | 95.7 |
| Gm09 | 46 843 750 | 3 092 | 95.8 |
| Gm10 | 50 969 635 | 3 127 | 96.1 |
| Gm11 | 39 172 790 | 2 060 | 96.1 |
| Gm12 | 40 113 140 | 1 723 | 95.3 |
| Gm13 | 44 408 971 | 3 337 | 95.9 |
| Gm14 | 49 711 204 | 3 106 | 95.6 |
| Gm15 | 50 939 160 | 4 252 | 96.3 |
| Gm16 | 37 397 385 | 3 052 | 95.9 |
| Gm17 | 41 906 774 | 2 871 | 95.8 |
| Gm18 | 62 308 140 | 5 213 | 96.3 |
| Gm19 | 50 589 441 | 3 344 | 95.9 |
| Gm20 | 46 773 167 | 3 092 | 96.1 |

**S2 Table. Overall accuracy of genotypic data following GBS analysis and imputation of missing data for all 20 soybean chromosomes.** Missing data were imputed using BEAGLE at MaxMD=80% and MinMAF=0.003.
