# Supplementary material for: Scanning and Filling: Ultra-Dense SNP Genotyping Combining Genotyping-By-Sequencing, SNP Array and Whole-Genome Resequencing Data
Source: PLoS One. 2015 Jul 10;10(7):e0131533. doi: 10.1371/journal.pone.0131533 (PMC4498655; doi:10.1371/journal.pone.0131533)
Supplement: S3 Table — Genotype data (1.4M SNPs) for 22 resequenced soybean lines were used to impute genotypes at loci that were untyped in the GBS SNP dataset while a 23rd resequenced line (either Gaillard, Mandarin or OAC-Lakeview) was set aside for validation. The imputed genotypes were compared to the result of the resequencing in the validation line. Three different permutations of the data (each time leaving out a single resequenced line from the reference panel) were performed. (DOCX) [file pone.0131533.s003.docx]

|  |  |  | Untyped loci imputation accuracy (%) | | |
| --- | --- | --- | --- | --- | --- |
| Data set | Imputation method | Reference panel | Gaillard | Mandarin | OAC-Lakeview |
|  |  |  |  |  |  |
|  | **BEAGLE** |  |  |  |  |
| GBS + SoySNP50K | Beagle | WGS | 88.1 | 89.2 | 87.9 |
|  |  |  |  |  |  |
|  | **IMPUTE2** |  |  |  |  |
| GBS + SoySNP50K | pre-Phasing by SHAPIT2 | WGS | 91.8 | 92.4 | 91.5 |
|  |  |  |  |  |  |

**S3 Table. Imputation accuracy of genotypes at untyped loci using whole-genome sequence data as a reference panel.** Genotype data (1.4M SNPs) for 22 resequenced soybean lines were used to impute genotypes at loci that were untyped in the GBS SNP dataset while a 23rd resequenced line (either Gaillard, Mandarin or OAC-Lakeview) was set aside for validation. The imputed genotypes were compared to the result of the resequencing in the validation line. Three different permutations of the data (each time leaving out a single resequenced line from the reference panel) were performed.
